# Tn Antigen Expression Defines an Immune Cold Subset of Mismatch-Repair Deficient Colorectal Cancer

**DOI:** 10.3390/ijms21239081

**Published:** 2020-11-29

**Authors:** Takuro Matsumoto, Hirokazu Okayama, Shotaro Nakajima, Katsuharu Saito, Hiroshi Nakano, Eisei Endo, Koji Kase, Misato Ito, Naoto Yamauchi, Leo Yamada, Yasuyuki Kanke, Hisashi Onozawa, Shotaro Fujita, Wataru Sakamoto, Motonobu Saito, Zenichiro Saze, Tomoyuki Momma, Kosaku Mimura, Koji Kono

**Affiliations:** 1 Department of Gastrointestinal Tract Surgery, Fukushima Medical University School of Medicine, Fukushima 960-1295, Japan; tak0912@fmu.ac.jp (T.M.); shotaro@fmu.ac.jp (S.N.); k-yamame@fmu.ac.jp (K.S.); nakano-h@fmu.ac.jp (H.N.); eisei-e@fmu.ac.jp (E.E.); kase55@fmu.ac.jp (K.K.); m-saku12@fmu.ac.jp (M.I.); yamato@fmu.ac.jp (N.Y.); yamada-r@fmu.ac.jp (L.Y.); kanke33@fmu.ac.jp (Y.K.); hisa444@fmu.ac.jp (H.O.); newyork@fmu.ac.jp (S.F.); ws1024@fmu.ac.jp (W.S.); moto@fmu.ac.jp (M.S.); z-saze@fmu.ac.jp (Z.S.); tmomma@fmu.ac.jp (T.M.); kmimura@fmu.ac.jp (K.M.); kojikono@fmu.ac.jp (K.K.); 2 Department of Medical Electrophysiology, Fukushima Medical University School of Medicine, Fukushima 960-1295, Japan; 3 Department of Blood Transfusion and Transplantation Immunology, Fukushima Medical University School of Medicine, Fukushima 960-1295, Japan

**Keywords:** colorectal cancer, deficient mismatch-repair, Tn antigen, immunotherapy, immune checkpoint

## Abstract

Colorectal cancer (CRC) cells often express Tn antigen, a tumor-associated truncated immature O-glycan (GalNAcα-O-Ser/Thr) that can promote tumor progression. Immunotherapies against Tn antigen have been developed and are being evaluated in clinical trials. Tn antigen can also be considered a novel immune checkpoint that induces immunosuppressive signaling through glycan-biding lectins to lead effector T cell apoptosis. We evaluated the correlation of Tn antigen expression by immunohistochemistry with mismatch-repair (MMR) status, tumor-infiltrating lymphocytes, tumor cell PD-L1 expression, and clinicopathological characteristics in 507 CRC patients. Although 91.9% of CRCs showed negative or weak Tn antigen staining (Tn-negative/weak), we identified a small subset of CRCs (8.1%) that displayed particularly intense and diffuse distribution of Tn antigen immunoreactivity (Tn-strong) that closely related to deficient MMR (dMMR). Moreover, 40 dMMR CRCs were stratified into 24 Tn-negative/weak dMMR tumors (60.0%) exhibiting dense CD8+ lymphocyte infiltrate concomitant with a high rate of PD-L1 positivity, and 16 Tn-strong dMMR tumors (40.0%) that demonstrated CD8+ T cell exclusion and a lack of PD-L1 expression, which was comparable to those of proficient MMR. Our finding suggests that the immune cold subset of patients with Tn-strong dMMR CRC may be effectively treated with immune checkpoint blockade therapy or cellular immunotherapy targeting Tn antigen.

## 1. Introduction

Colorectal cancer (CRC) develops through the accumulation of various genetic and epigenetic alterations. The majority of CRCs (~85%) exhibit chromosomal instability, whereas about 15% of CRCs with deficient mismatch-repair (dMMR) are susceptible to mutations in repetitive DNA sequences (microsatellites), resulting in high-level microsatellite instability (MSI-H) [1,2,3]. Such molecular features can affect both cancer cell behavior and the creation of the tumor microenvironment (TME), thereby correlating with individual patient prognosis and therapeutic response [2,4]. In early stage patients, dMMR is associated with a low risk of recurrence and a lack of treatment benefit from adjuvant chemotherapy [2]. Patients with dMMR metastatic CRC are generally less responsive to conventional chemotherapy, and have poorer survival outcomes than those with proficient mismatch-repair (pMMR) or microsatellite stable (MSS) CRC [5,6,7]. Importantly, dMMR CRCs are heavily infiltrated by tumor-infiltrating lymphocytes (TILs), thus, they are generally considered to be immunologically hot tumors [3,5,8]. To evade immune-mediated killing in this T cell-inflamed (hot) TME, dMMR cancer cells express T cell inhibitory ligands, such as PD-L1, on their surface, which bind to co-inhibitory receptors, such as PD1 on T cells [3,8]. Recently, immunotherapy with immune checkpoint inhibitors (ICIs) against PD1/PD-L1 signaling has generated great excitement because of its success in achieving long-term durable responses in patients with metastatic CRC whose tumors are dMMR, in which ICIs antagonize T cell inhibitory signaling, potentiating cytotoxic killing of tumor cells [5,6,8,9,10]. In the current practice, MMR/MSI testing has a strong predictive value for the use of ICIs in metastatic CRC [2,5,9]. However, the response rate to the current ICIs ranged from 30% to 60% in dMMR CRCs, which was associated neither with the expression of PD-L1 nor mutations in *KRAS* and *BRAF* [2,5,8,9,10]. Therefore, one of the major challenges is to identify biomarker-driven patient subsets among the heterogeneous spectrum of dMMR CRC who could be effectively treated with combined or more targeted immunotherapeutic strategies.

Cancer cells express aberrant glycan structures on their surface, namely, tumor-associated carbohydrate antigens (TACAs) that can promote tumor progression and metastasis, often correlating with poor prognosis [11]. Most TACAs are overexpressed in premalignant and malignant tissues, but found in low amounts in their normal counterparts. Indeed, some TACAs are utilized as serological biomarkers for cancer detection (e.g., CA19-9) [11,12]. One of the most prevalent TACAs in cancer is Tn antigen (GalNAcα-O-Ser/Thr), a truncated immature O-glycan formed from an incomplete synthesis mechanism, by which normal glycan elongation is impaired during malignancy [11,12,13]. Tn antigen has been considered a promising target for therapeutic vaccination and antibody immunotherapy [14]. Moreover, engineered chimeric antigen receptor (CAR) T cells against Tn antigen on MUC1 (Tn-MUC1) has recently been developed in solid tumors [15]. Such immunotherapeutic strategies targeting Tn antigen are currently being evaluated in clinical trials. It is also worth noting that altered glycosylation can not only promote tumor progression, but induce immunosuppressive signaling through glycan-binding receptors (lectins) expressed by a variety of immune cells. It has thus recently been proposed that specific glycans, such as Tn antigen, found on tumor cells, referred to as the “glyco-code”, can be considered as a novel immune checkpoint, offering new immunotherapeutic opportunities [16,17,18]. In the TME, Tn antigen abrogates Th1 cell responses and stimulates T cells to produce interleukin-17 (IL-17), likely favoring immune escape of tumor cells [19]. Moreover, Tn antigen on tumor cells interact with macrophage galactose-specific lection (MGL) on antigen-presenting cells, driving an immune inhibitory signaling by increasing anti-inflammatory interleukin-10 (IL-10) production and inducing effector T cell apoptosis [16,20,21]. Correspondingly, in vivo tumor growth was driven by overexpressed Tn antigen on a genetically modified CRC cell line in a mouse model, accompanied with reduced levels of CD8+ T cell infiltration [22]. Therefore, Tn antigen could also be targeted as an immune checkpoint by preventing its interaction with inhibitory immune receptors [16]. Since dMMR CRCs represent a promising candidate for treatment with immunotherapy, further evaluation of Tn antigen expression in CRC is needed to facilitate precise immunotherapeutic approaches. However, no studies have addressed the association of the expression of Tn antigen with MMR status and the immunophenotypes in CRC. In this study, we conducted immunohistochemistry for Tn antigen using a large cohort of CRC to investigate the association of the expression of Tn antigen with clinicopathological and molecular features, including MMR status, tumor infiltrating lymphocytes, and PD-L1 expression.

## 2. Results

### 2.1. Tn Antigen Expression in CRC

**Figure 1:**
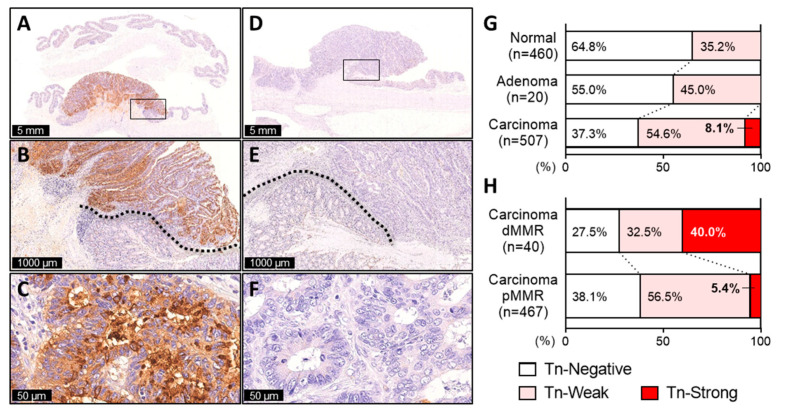
Immunohistochemistry for Tn antigen identifies a Tn-strong subset of colorectal cancer associated with deficient mismatch-repair (dMMR). (**A**–**C**) Representative images of a resected specimen of dMMR tubular adenocarcinoma showing diffuse and intense distribution of Tn antigen immunoreactivity (Tn-strong) in the tumor, but not in adjacent non-tumor mucosa. (**D**–**F**) Representative images of a resected specimen of proficient mismatch-repair (pMMR) tubular adenocarcinoma showing Tn-negative expression. (**G**,**H**) Relative proportion of Tn-negative, Tn-weak, and Tn-strong in non-tumor mucosa, adenoma, and carcinoma samples (**G**) or in dMMR and pMMR carcinoma samples (**H**).

We conducted immunohistochemistry for Tn antigen using surgically resected whole tissue specimens, including 20 adenomas and 507 primary CRCs, in which 460 adjacent non-tumor mucosa were also available for evaluation. Immunoreactivity for Tn antigen staining in the cytoplasm and cell membrane were respectively evaluated and then combined to obtain the Tn score, as described in Supplementary Figure S1. In tumor adjacent mucosa, non-neoplastic epithelial cells often displayed weak to moderate granular staining predominantly in the supranuclear cytoplasm, but membranous staining was undetectable (Supplementary Figure S1 and Figure 1A–F). The staining patterns of Tn antigen in adenomas were similar to those of non-tumor mucosa. We observed 35.2% of non-tumor mucosa, and 45.0% of adenomas were positive for Tn antigen expression (Figure 1G). By contrast, in CRC tissues, the cytoplasmic and membranous expression of Tn antigen was observed in cancer cells with considerably varying degrees of staining intensity and its extent, occasionally accompanied by staining in the extracellular mucin deposit (Supplementary Figure S1–S3 and Figure 1A–F). Of the 507 CRC tissues, 189 (37.3%) were defined as Tn-negative (Tn score 0–3) and 318 (62.7%) were defined as Tn-positive (Tn score 4–24), and the latter was further classified into 277 Tn-weak (Tn score 4–15) (54.6%) and 41 Tn-strong (Tn score 16–24) (8.1%) tumors (Figure 1G). In Tn-strong tumors, intense cytoplasmic and membranous staining was diffusely distributed throughout the tumor (Supplementary Figure S2 and Figure 1A–C).

### 2.2. A Small Subset of CRC Exhibiting Diffuse and Intense Tn Staining

We next sought to determine clinicopathological characteristics of CRCs according to Tn antigen expression by focusing particularly on the small subset of tumors exhibiting Tn-strong. To this end, we combined Tn-negative and Tn-weak tumors, in which there was no significant difference in clinicopathological characteristics except for mucinous histology, between tumors with Tn-negative and Tn-weak (Supplementary Table S1). As shown in Table 1, compared with Tn-negative/weak, Tn-strong tumors were significantly associated with proximal tumor location (*p* = 0.004), poor differentiation (*p* < 0.0001), mucinous histology (*p* < 0.0001), and deeper depth of invasion (*p* = 0.050). Nonetheless, Tn-strong was not correlated with age, gender, lymphatic invasion, venous invasion, lymph node metastasis, distant metastasis, or stage of disease (*p* > 0.05). Strikingly, we found a significant association between Tn-strong and dMMR (*p* < 0.0001, Table 1), in which 39.0% of Tn-strong tumors were dMMR, whereas almost all (approximately 95%) tumors showing Tn-negative/weak were pMMR. No association was found in terms of CD8+, CD4+, and Foxp3+ lymphocyte infiltration or tumor cell PD-L1 expression between tumors with Tn-negative/weak and Tn-strong (*p* > 0.05, Table 1).

### 2.3. Tn-Strong dMMR Tumors Showed Immune Cold Characteristics

**Figure 2:**
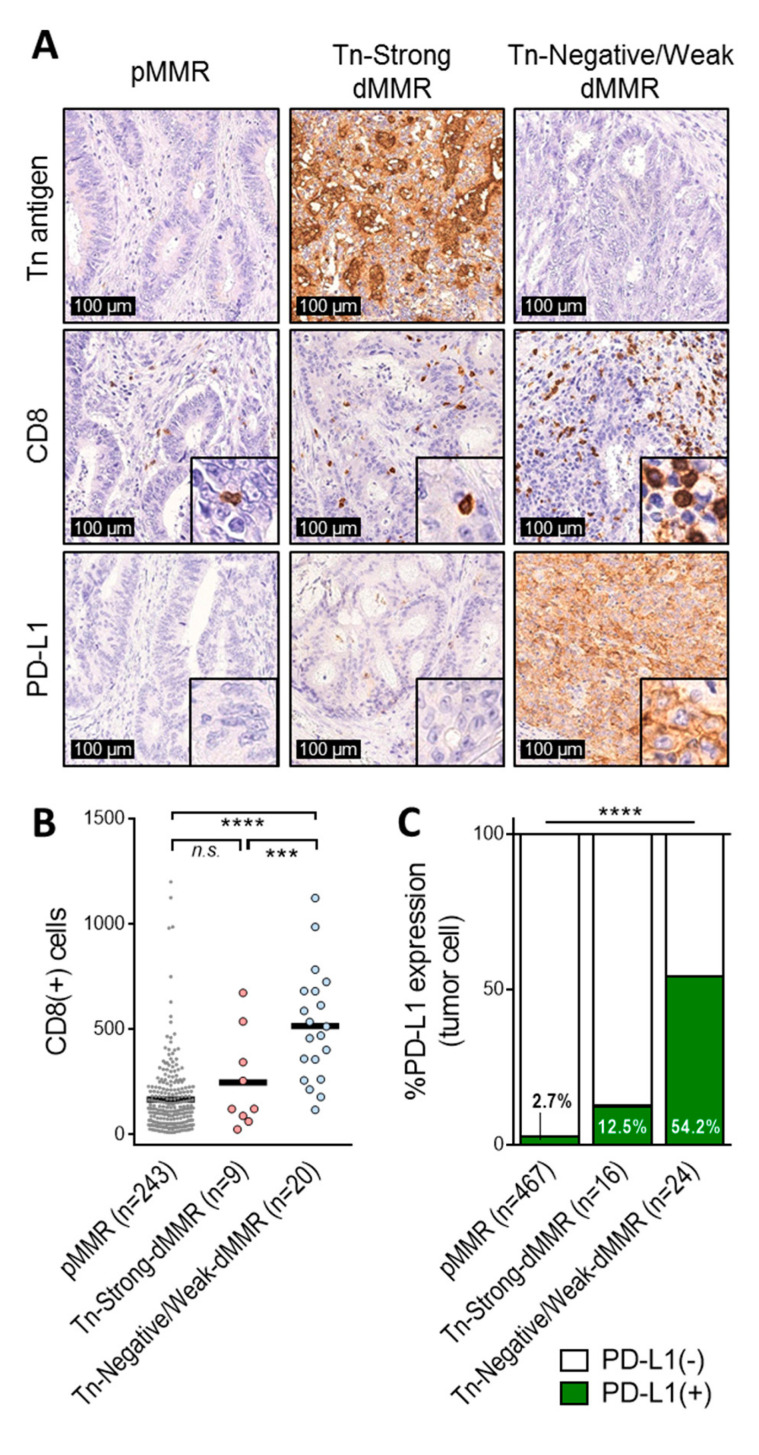
The Tn-strong dMMR subset lacks common immunological characteristics of dMMR tumors, including CD8+ T cell infiltration and tumor cell surface PD-L1 expression. (**A**) Representative immunohistochemistry images for Tn antigen, CD8, and PD-L1 in pMMR, Tn-strong dMMR, and Tn-negative/weak dMMR tumors. (**B**,**C**) Tn-strong dMMR tumors displaying lower levels of CD8+ T cell infiltration (**B**) and PD-L1 expression (**C**), compared to those of Tn-negative/weak dMMR tumors. **** *p* < 0.0001, *** *p* < 0.001, *n.s. p* > 0.05.

The finding described above prompted us to further investigate clinicopathological and immune profiles by stratifying dMMR tumors according to the expression of Tn antigen. Among 40 dMMR CRCs, two distinct subgroups were defined, including 16 Tn-strong dMMR tumors (40.0%) and 24 Tn-negative/weak dMMR tumors (60.0%) (Figure 1H). However, as shown in Table 2, we found nearly identical clinicopathological features between the two groups, such as tumor differentiation, mucinous histology, tumor invasion, lymph node metastasis, and distant metastasis (*p* > 0.05). By contrast, tumor cell PD-L1 expression was significantly frequently observed in Tn-negative/weak dMMR (54.2% were positive for PD-L1) compared to that of Tn-strong dMMR tumors (12.5% were positive for PD-L1) (*p* = 0.010). Moreover, significantly higher levels of CD8+ T cell infiltrate were demonstrated in Tn-negative/weak dMMR tumors than that of Tn-strong dMMR (*p* = 0.014), although no difference was found in CD4+ or Foxp3+ cell infiltration between the two groups (*p* > 0.05). Representative images for Tn antigen, CD8, and PD-L1 staining in pMMR, Tn-strong dMMR, and Tn-negative/weak dMMR tumors are demonstrated in Figure 2A. Notably, high levels of CD8+ TILs, along with a high incidence of PD-L1 positivity, but not with CD4+ TILs or Foxp3+ TILs, were specifically observed in Tn-negative/weak dMMR tumors, but not in pMMR or Tn-strong dMMR tumors (Figure 2B,C and Supplementary Figure S4). On the other hand, CD8+ TILs and PD-L1 expression in Tn-strong dMMR tumors were comparable to that of pMMR tumors (Figure 2B,C). When only pMMR tumors were analyzed, the expression of Tn antigen appeared to have no significant impact on the levels of CD8+ TILs, CD4+ TILs, Foxp3+ TILs, or PD-L1 expression (Supplementary Figure S5).

## 3. Discussion

Despite the initial failure of ICIs in pMMR CRC, it has been established that patients with dMMR CRC represent a biomarker-defined subgroup that contains potentially good responders to immunotherapy with ICIs, however, substantial clinical and molecular diversity still exists within this population that may affect ICI treatment response. Indeed, nearly half of metastatic dMMR CRC cases exhibit primary resistance to the current ICIs, potentially due to multiple tumor escape mechanisms [2,8]. Since immunotherapies are expected to be potentiated by combinatorial strategies to overcome immunosuppressive mechanisms, recent advances in the detailed understanding of the TME in CRC have attempted to further stratify dMMR tumors into different subsets, with the final goal of defining the eligibility of patients with dMMR CRC for more personalized immunotherapeutic interventions. For example, higher tumor mutation burden (TMB), CD8+ lymphocyte density, and TIL count might be predictive of a good response to ICIs in dMMR CRCs [10,23,24,25]. Several lines of evidence have demonstrated that TGFβ-rich cancer stroma is considered a determinant of immune exclusion, worse prognosis, and poor response to ICIs, suggesting that the combined blockade of TGFβ-signaling and immune checkpoints offers a promising strategy for patients with pMMR, as well as in a subset of dMMR CRCs [8,26,27,28]. Mutations in *JAK1/2* and losses of *B2M* in dMMR CRCs may also be potential mechanisms of ICI resistance [8,24,29,30]. However, no predictive biomarkers have so far been approved for clinical application in patients with dMMR CRCs.

Tn antigen is the only precursor for O-glycans, and this mucin-type O-glycosylation is initiated by a family of 20 ppGalNAc-Ts to form Tn antigen, and, in turn, T-synthase (C1GalT1) with its molecular chaperone Cosmc (C1GalT1C1) converts Tn antigen to the core 1 O-glycan elongation, which are further elongated, branched, and capped by a large number of glycosyltransferases [11,31]. The overexpression of Tn antigen can result mainly from inactivation of Cosmc due to somatic mutation or epigenetic silencing in several malignancies, including CRC [11,14]. In addition, altered expression or localization of different ppGalNAc-Ts can regulate the expression of Tn antigen [31]. Our previous work reported that ppGalNAc-T6 was frequently downregulated via epigenetic silencing in dMMR CRCs compared to those of pMMR, suggesting that it can at least in part contribute to the overexpression of Tn antigen [13]. Repeated DNA sequences in the Cosmc gene might be susceptible to microsatellite instability [32]. We thus speculate that several intrinsic mechanisms are involved in the upregulation of cell surface Tn antigen in dMMR CRCs.

The expression of Tn antigen in CRC has been studied since more than three decades ago. Earlier studies analyzed a relatively small number of specimens (less than 30 CRC tissues), and revealed that 72–82% of CRCs were found to be positive for Tn antigen expression [33,34,35]. Oshikiri et al. reported that 68 of 146 CRCs (46.6%) were Tn positive [36]. More recently, Jiang et al. examined 186 CRC specimens, and 161 (86.6%) were determined to be positive for Tn antigen [37]. Although the Tn antigen positive rate varied among studies likely due to the detection approaches, including different monoclonal antibodies or lectins used, the expression pattern and the localization of Tn staining in CRC was highly consistent. In the present study, in a large cohort of CRC (*n* = 507), using a monoclonal antibody MLS128 for immunohistochemistry, we again confirmed that the majority (62.7%) of CRCs were positive for Tn antigen. Nevertheless, unlike previous studies described above, we herein identified a small subset of CRCs (8.1%) displaying strikingly intense and diffuse distributions of Tn antigen expression. Although none of the previous studies addressed the correlation between Tn antigen and MMR status, we found that the Tn-strong tumors were highly enriched within dMMR CRCs, where 16 of 40 (40.0%) of dMMR tumors were determined to be Tn-strong, which was in clear contrast to only 5.4% of pMMR tumors showing Tn-strong. More noteworthy is the fact that the unique subset of Tn-strong dMMR CRCs specifically lacked common immunological characteristics of dMMR CRC, such as dense CD8+ T cell infiltrate in the TME and PD-L1 expression on tumor cells. High levels of TILs have been considered not only an indicator of host immune response to the tumor, but also a favorable prognostic marker in CRC, independent of MMR status [38]. Particularly, in early stage patients with dMMR CRC, the pronounced anti-tumor immune response characterized by an increased density of intratumoral T cells seems to explain their generally good prognosis. A recent report demonstrated that a high number of TILs, along with high TMB, was correlated with clinical responses and survival benefit in patients with dMMR CRCs who were treated with ICIs [25]. By contrast, unresponsiveness to ICI treatment in pMMR CRCs is likely associated with low TMB and the lack of immune infiltration due to low tumor neoantigens. Our finding therefore suggests that Tn antigen overexpression is one of the underlying mechanisms of T cell exclusion in dMMR CRC. Although this study lacks direct assessment of the predictive role of Tn-strong for the efficacy of ICIs in patients with dMMR CRC, the Tn-strong dMMR subset may represent an immune cold subgroup of patients who do not respond well to the current immunotherapeutic strategies. Future studies would be required to address the prognostic as well as predictive roles of Tn-strong expression, particularly in patients with metastatic dMMR CRC who are treated with ICIs.

Tn antigen has been prioritized for the development of anti-cancer vaccines [39,40]. In a Phase 1/2 trial of human prostate cancer, Tn-MUC1 DC vaccination was able to induce a significant CD8+ T cell response [40]. In a recent preclinical mouse model, Tn antigen mimetic vaccination elicited a robust immune response and delayed tumor progression [41]. Most recently, the fully synthetic glycopeptide MAG-Tn3 therapeutic vaccine was designed to induce antibody responses against Tn antigen, and was evaluated in a phase 1 clinical trial (NCT02364492) [42]. Other strategies to target Tn antigen include cellular immunotherapy and antibody immunotherapy. Cellular immunotherapy with engineered CAR T cells against Tn antigen on MUC1 (Tn-MUC1) has been developed, and is being evaluated in a phase 1 clinical trial in patients with solid cancers (NCT04025216) [15,18,43,44]. Notably, Tn antigen has been proposed to be considered an immune checkpoint [16], as Tn antigen can induce immune suppression and effector T cell apoptosis, likely through glycan-biding lectins and increased anti-inflammatory mediators [19,20,21]. Correspondingly, a Cosmc-deleted CRC cell line model expressing high levels of cell surface Tn antigen not only exhibited decreased levels of gene signatures related to cytotoxic T cell activation in vitro, but also promoted in vivo tumorigenesis correlated with CD8+ T cell exclusion [22]. These data consistently suggest that antibody immunotherapy blocking the interactions of Tn antigen with inhibitory immune receptors may also serve as a promising immunotherapeutic strategy. We thus propose that the Tn-strong dMMR subset of CRC exhibiting T cell exclusion may be a good candidate for immune checkpoint blockade immunotherapy targeting Tn antigen as well as CAR T cell therapy against Tn antigen. Further preclinical and clinical investigation is clearly needed to elucidate the diagnostic and therapeutic values of Tn antigen in CRC.

In conclusion, we identified a distinct subgroup of dMMR CRC exhibiting strong Tn antigen expression that is characterized by CD8+ T cell exclusion and a lack of PD-L1 expression, suggesting that Tn antigen is predictive of poor response to ICIs in dMMR CRCs. Patients with Tn-strong dMMR CRC may be effectively treated with immune checkpoint blockade therapy or cellular immunotherapy targeting Tn antigen.

## 4. Materials and Methods

### 4.1. Patient Samples

We enrolled 20 patients with colon adenoma and 507 patients with stage 0 to IV primary CRC who underwent surgical resection at Fukushima Medical University Hospital between 2002 and 2013 without preoperative chemotherapy or radiotherapy. Their available formalin-fixed paraffin-embedded (FFPE) whole tissue sections were used for immunohistochemistry. Clinical information was retrospectively obtained from medical records. The study was conducted in accordance with the Declaration of Helsinki and was approved by the Institutional Review Board of Fukushima Medical University.

### 4.2. Immunohistochemistry

Four-µm thick sections were deparaffinized in xylene and rehydrated in a series of ethanol. Endogenous peroxidases were blocked with 0.3% hydrogen peroxide in methanol. Antigens were retrieved by autoclave, and slides were incubated with the following primary antibodies: CD4 (mouse; clone 4B12; M7310, Dako/Agilent Technologies, Santa Clara, CA, USA; 1:100), CD8 (mouse; clone C8/144B; M7103; Dako/Agilent Technologies; 1:100), Foxp3 (mouse; clone 236A/E7; ab20034; abcam, Cambridge, UK; 1:200), PD-L1 (rabbit; clone E1L3N; 13684; Cell Signaling Technology, Danvers, MA, USA; 1:400), and Tn antigen (mouse; clone MLS128; Wako, Osaka, Japan; 1:500). Sections were subsequently incubated with horseradish peroxidase (HRP)-coupled anti-mouse or anti-rabbit secondary antibodies (Envision + System, K4003 or K4001; Dako/Agilent Technologies). Peroxidase was visualized with diaminobenzidine (DAB; Dojindo, Kumamoto, Japan), and nuclei were counterstained with Mayer’s Hematoxylin Solution (131-09665; Wako/Fujifilm, Osaka, Japan). Negative controls were done by replacing primary antibodies with PBS.

### 4.3. Assessment of Staining

For Tn antigen, staining in the cytoplasm and cell membrane, respectively, was evaluated for cytoplasmic Tn score and membranous Tn score. The intensity of staining was graded as follows: negative (0), weak (1), moderate (2), or strong (3), and the percentage of positive cells was graded as follows: 0% (0), 1–25% (1), 26–50% (2), 51–75% (3), or 76–100% (4). The intensity and the positivity scores for staining in the cytoplasm and cell membrane were multiplied to obtain the cytoplasmic Tn score (0–12) and membranous Tn score (0–12), respectively, and then they were combined to obtain the total Tn score, representing overall Tn antigen expression levels ranging from 0 to 24. Tn score in each section was further classified into Tn-negative (0–3), Tn-weak (4–15), or Tn-strong (16–24). Assessment of Tn antigen expression is summarized in Supplementary Figure S1. Evaluation of CD4 and CD8 staining was described elsewhere [27]. Briefly, the invasive front region of the tumor was reviewed in four independent areas, and evaluated by counting the number of stained lymphocytes at a magnification of 400×. For Foxp3 staining, four independent hot spot areas were selected at a magnification of 40×, and then counted at a magnification of 400×, as described previously [27]. For PD-L1 staining, specimens were considered PD-L1 positive when more than 5% of the tumor cells showed membranous staining of any intensity with or without cytoplasmic staining, as described previously [45]. The immunostains were evaluated by four observers (K.S, L.Y, E.E, and T.M) who were blinded from all of the clinical data.

### 4.4. Determination of MMR Status

Immunohistochemistry for MMR proteins (MLH1, MSH2, MSH6, and PMS2) was performed as described previously [13]. Loss of at least one MMR protein was defined as dMMR, and tumors with intact MMR protein expression were defined as pMMR.

### 4.5. Statistical Analysis

Fisher’s exact test, the χ2 test, unpaired *t*-test, or the Mann–Whitney U test were used to determine differences between two variables where appropriate. Comparison of variables across the three groups was assessed using one-way ANOVA with the Turkey post hoc test. All statistical analyses were conducted using GraphPad Prism v6.04 (Graphpad Software Inc., San Diego, CA, USA) or SPSS Statistics version 26 (IBM Corporation, NY, USA). All *p*-values were two-sided, and *p*-values less than 0.05 were considered statistically significant.

## Figures and Tables

**Table 1:** Clinicopathological characteristics of colorectal cancer patients according to Tn antigen expression.

		Total (*n* = 507)	Tn-Negative/Weak	Tn-Strong	*p*-Value
			*n* = 466 (91.9%)	*n* = 41 (8.1%)	
Age					0.233
	Mean ± SD	68.4 ± 11.6	68.2 ± 11.4	70.5 ± 13.3	
Gender					0.315
	Male	319	290 (62.2%)	29 (70.7%)	
	Female	188	176 (37.8%)	12 (29.3%)	
Location					0.004
	Proximal colon	193	168 (36.1%)	25 (61.0%)	
	Distal colon	133	126 (27.0%)	7 (17.1%)	
	Rectum	181	172 (36.9%)	9 (22.0%)	
Tumor differentiation					<0.0001
	Well-Moderately	478	447 (95.9%)	31 (75.6%)	
	Poorly	29	19 (4.1%)	10 (24.4%)	
Histology					<0.0001
	Non-mucinous	482	451 (96.8%)	31 (75.6%)	
	Mucinous	25	15 (3.2%)	10 (24.4%)	
Tumor invasion					0.050
	Tis	32	30 (6.4%)	2 (4.9%)	
	T1	61	59 (12.7%)	2 (4.9%)	
	T2	73	69 (14.8%)	4 (9.8%)	
	T3	194	177 (38.0%)	17 (41.5%)	
	T4	147	131 (28.1%)	16 (39.0%)	
Lymphatic invasion					0.858
	Absent	147	136 (29.2%)	11 (26.8%)	
	Present	360	330 (70.8%)	30 (73.2%)	
Venous invasion					1.000
	Absent	129	119 (25.5%)	10 (24.4%)	
	Present	378	347 (74.5%)	31 (75.6%)	
Lymph node metastasis					0.395
	Absent	316	288 (61.8%)	28 (68.3%)	
	Present	188	176 (37.8%)	12 (29.3%)	
	Not available	3	2 (0.4%)	1 (2.4%)	
Distant metastasis					0.822
	Absent	428	394 (84.5%)	34 (82.9%)	
	Present	79	72 (15.5%)	7 (17.1%)	
Stage					0.544
	0	31	29 (6.2%)	2 (4.9%)	
	I	111	106 (22.7%)	5 (12.2%)	
	II	153	135 (29.0%)	18 (43.9%)	
	III	133	124 (26.6%)	9 (22.0%)	
	IV	79	72 (15.5%)	7 (17.1%)	
PD-L1 expression on tumor cells					0.487
	Negative	479	441 (94.6%)	38 (92.7%)	
	Positive	28	25 (5.4%)	3 (7.3%)	
MMR status					<0.0001
	pMMR	467	442 (94.8%)	25 (61.0%)	
	dMMR	40	24 (5.2%)	16 (39.0%)	
CD8+ cells					0.432
	Mean ± SD	194.7 ± 201.2	197.6 ± 204.1	163.1 ± 167.1	
CD4+ cells					0.432
	Mean ± SD	96.0 ± 96.5	97.4 ± 99.1	80.8 ± 61.3	
Foxp3+ cells					0.967
	Mean ± SD	386.9 ± 221.6	386.7 ± 225.7	388.5 ± 187.9	

**Table 2:** Clinicopathological characteristics of patients with mismatch-repair deficient colorectal cancer according to Tn antigen expression.

		Tn-Negative/Weak dMMR	Tn-Strong dMMR	*p*-Value
		*n* = 24 (60.0%)	*n* = 16 (40.0%)	
Age				0.101
	Mean ± SD	63.4 ± 15.8	71.6 ± 13.7	
Gender				1.000
	Male	11 (45.8%)	8 (50.0%)	
	Female	13 (54.2%)	8 (50.0%)	
Location				0.062
	Proximal colon	15 (62.5%)	14 (87.5%)	
	Distal colon	4 (16.7%)	2 (12.5%)	
	Rectum	5 (20.8%)	0 (0.0%)	
Tumor differentiation				0.729
	Well-Moderately	16 (66.7%)	12 (75.0%)	
	Poorly	8 (33.3%)	4 (25.0%)	
Histology				1.000
	Non-mucinous	21 (87.5%)	14 (87.5%)	
	Mucinous	3 (12.5%)	2 (12.5%)	
Tumor invasion				0.916
	T1	0 (0.0%)	1 (6.3%)	
	T2	7 (29.2%)	2 (12.5%)	
	T3	10 (41.7%)	10 (62.5%)	
	T4	7 (29.2%)	3 (18.8%)	
Lymphatic invasion				0.729
	Absent	8 (33.3%)	4 (25.0%)	
	Present	16 (66.7%)	12 (75.0%)	
Venous invasion				1.000
	Absent	5 (20.8%)	4 (25.0%)	
	Present	19 (79.2%)	12 (75.0%)	
Lymph node metastasis				1.000
	Absent	17 (70.8%)	10 (62.5%)	
	Present	7 (29.2%)	5 (31.3%)	
	Not available	0 (0.0%)	1 (6.3%)	
Distant metastasis				0.553
	Absent	23 (95.8%)	14 (87.5%)	
	Present	1 (4.2%)	2 (12.5%)	
Stage				0.471
	I	6 (25.0%)	3 (18.8%)	
	II	11 (45.8%)	7 (43.8%)	
	III	6 (25.0%)	4 (25.0%)	
	IV	1 (4.2%)	2 (12.5%)	
PD-L1 expression on tumor cells				0.010
	Negative	11 (45.8%)	14 (87.5%)	
	Positive	13 (54.2%)	2 (12.5%)	
CD8+ cells				0.014
	Mean ± SD	515.1 ± 265.5	247.3 ± 228.0	
CD4+ cells				0.443
	Mean ± SD	134.1 ± 114.9	101.8 ± 68.8	
Foxp3+ cells				0.129
	Mean ± SD	456.7 ± 214.4	353.3 ± 189.6	

## Supplementary Materials

Supplementary materials can be found at https://www.mdpi.com/1422-0067/21/23/9081/s1. Figure S1. Assessment of immunohistochemistry for Tn antigen expression. Figure S2. Representative cases of deficient mismatch-repair (dMMR) tumorsbshowingbTn-Strong expression (Case001–003). Figure S3. Representative cases of tumors showing Tn-Weak (Case004–005) or Tn-Negative (Case006–008) expression. Figure S4. CD4+ or Foxp3+ T cell infiltration in pMMR, Tn-Strong-dMMR and Tn-Negative/Weak-dMMR tumors. Figure S5. CD8+, CD4+ or Foxp3+ T cell infiltration in pMMR tumors, according to Tn antigen expression. Table S1. Clinicopathological characteristics of colorectal cancerpatients according to Tn antigen expression 384.8±.

## Abbreviations

CRC	colorectal cancer
dMMR	deficient mismatch-repair
pMMR	proficient mismatch-repair
MSI-H	high-level microsatellite instability
MSS	microsatellite stable
TME	tumor microenvironment
TIL	tumor-infiltrating lymphocyte
ICI	immune checkpoint inhibitors
TACA	tumor-associated carbohydrate antigen
FFPE	formalin-fixed paraffin-embedded
