# Supplementary material for: Tn Antigen Expression Defines an Immune Cold Subset of Mismatch-Repair Deficient Colorectal Cancer
Source: Int J Mol Sci. 2020 Nov 29;21(23):9081. doi: 10.3390/ijms21239081 (PMC7730766; doi:10.3390/ijms21239081)
Supplement: Supplementary file 1 [file ijms-21-09081-s001.pdf]

Supplementary Figure S1

Tn-Cytoplasmic stainig score = A x B (0-12)

(A) Intensity of the reaction		(B) Number of positive cells (%)	
0	No reaction	0	0%
1	Weak reaction	1	1-25%
2	Moderate reaction	2	26-50%
3	Strong reaction	3	51-75%
		4	76-100%

Tn-score (0-24) = Cytoplasmic + Membranous staining score

0-3	Tn-Negative
4-15	Tn-Weak (Tn-Positive)
16-24	Tn-Strong (Tn-Positive)

Tn-Membranous stainig score = C x D (0-12)

(C) Intensity of the reaction		(D) Number of positive cells (%)	
0	No reaction	0	0%
1	Weak reaction	1	1-25%
2	Moderate reaction	2	26-50%
3	Strong reaction	3	51-75%
		4	76-100%

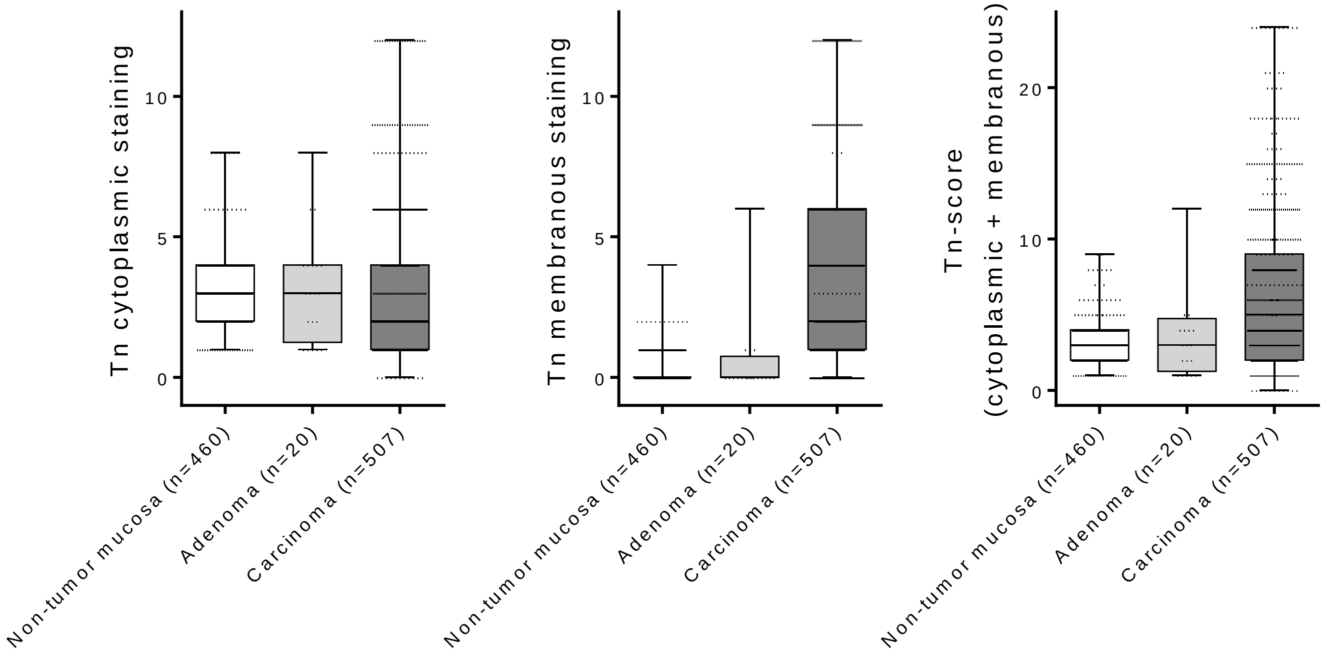

Supplementary Figure S1. Assessment of immunohistochemistry for Tn antigen expression. The intensity and the positivity scores for staining in cytoplasm and cell membrane were multiplied to obtain cytoplasmic Tn score (0-12) and membranous Tn score (0-12), respectively, and then they were combined to obtain total Tn score, representing overall Tn antigen expression levels ranging from 0 to 24.

Supplementary Figure S2

Case 001, dMMR, score 24 (Tn-Strong)

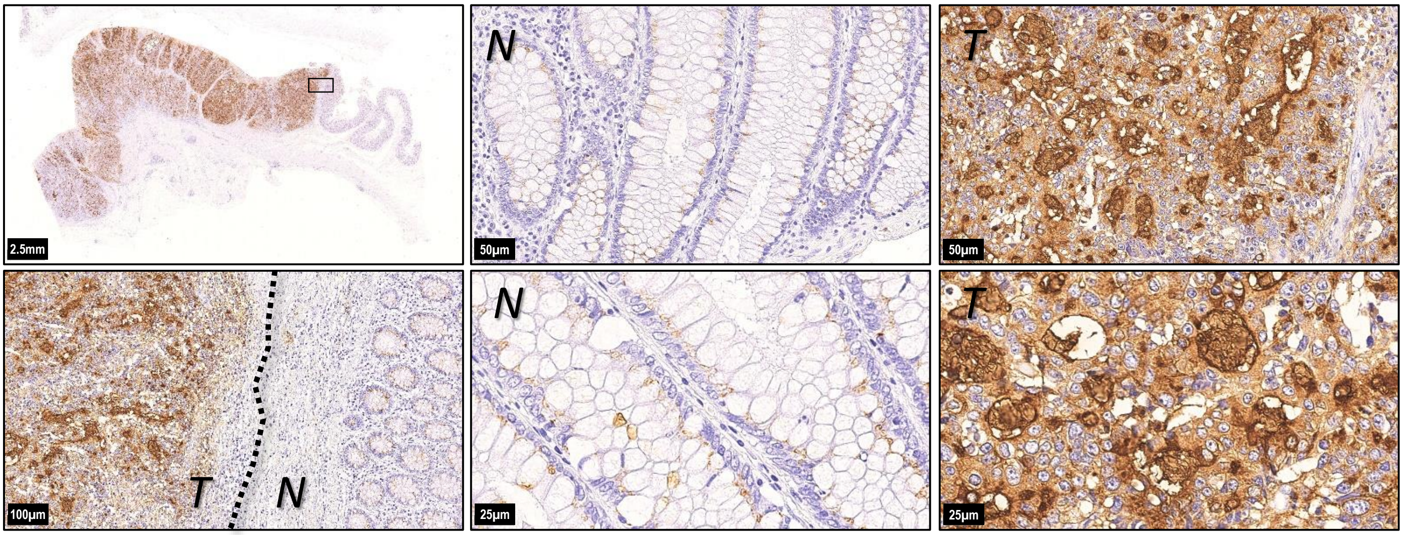

Case 002, dMMR, score 24 (Tn-Strong)

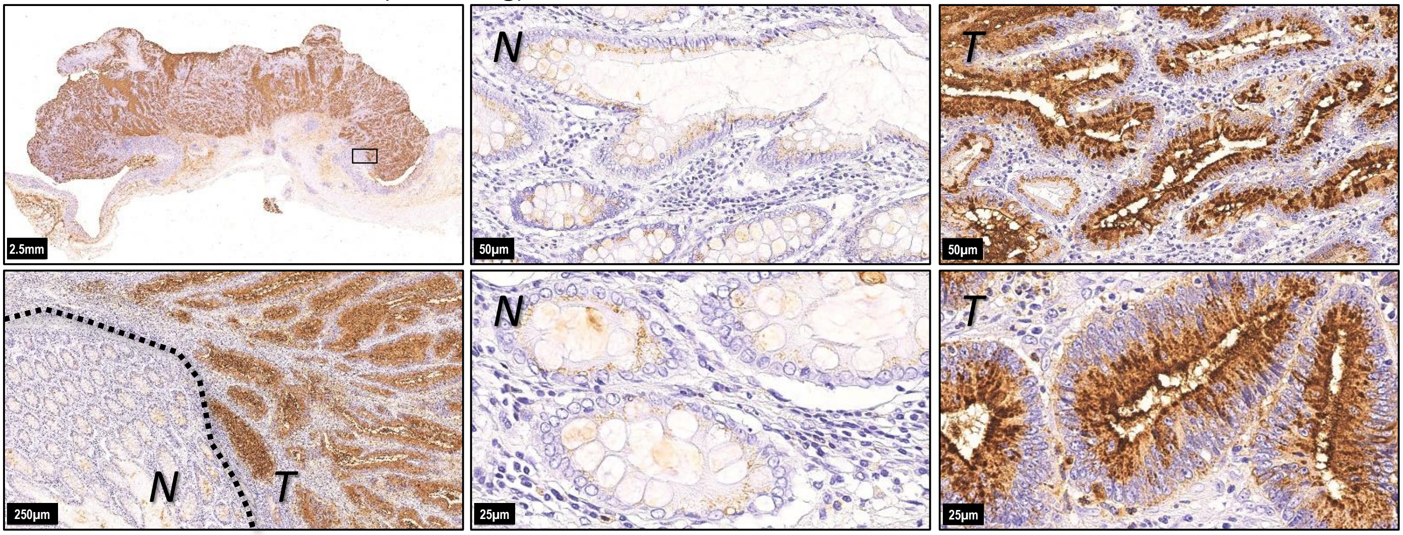

Case 003, dMMR, score 24 (Tn-Strong)

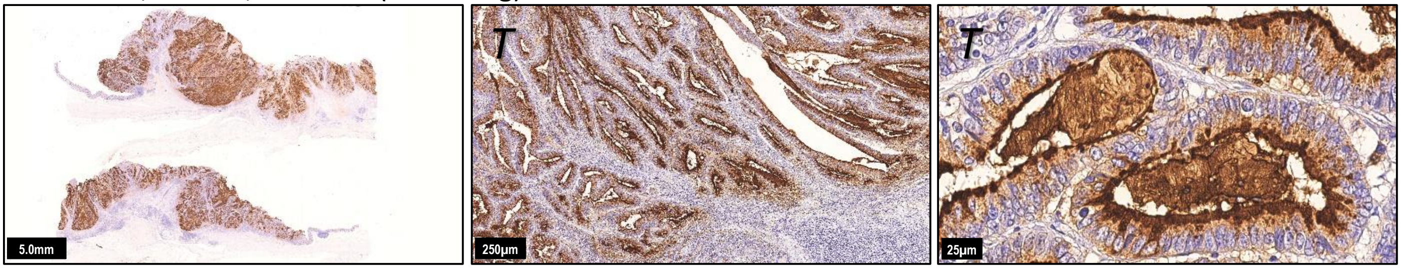

Supplementary Figure S2. Representative cases of deficient mismatch-repair (dMMR) tumors showing Tn-Strong expression (Case 001-003). Intense and diffuse Tn antigen immunoreactivity is found in tumor (T) but not in adjacent non-tumor mucosa (N).

Supplementary Figure S3

Case 004, pMMR, score 6 (Tn-Weak)

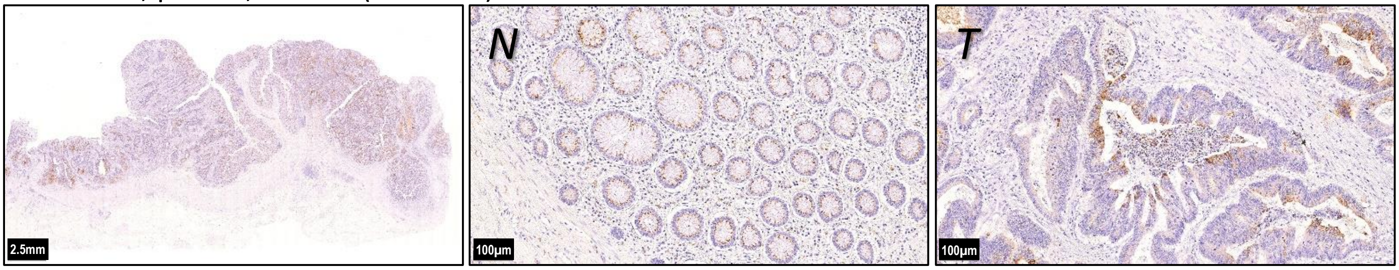

Case 005, pMMR, score 4 (Tn-Weak)

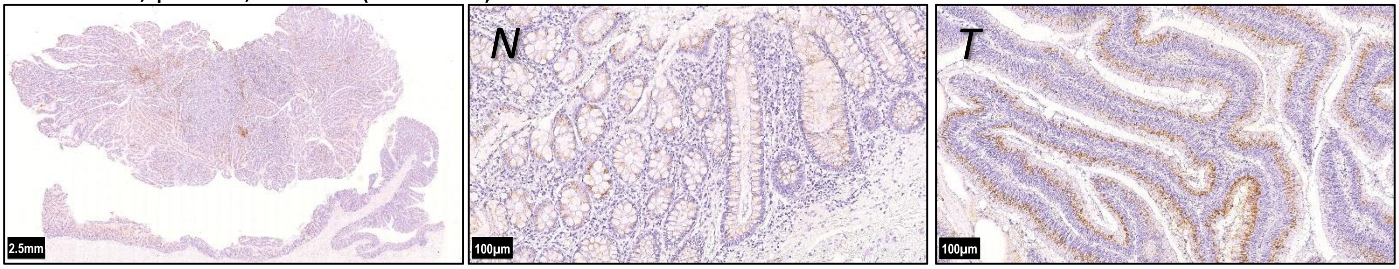

Case 006, pMMR, score 1 (Tn-Negative)

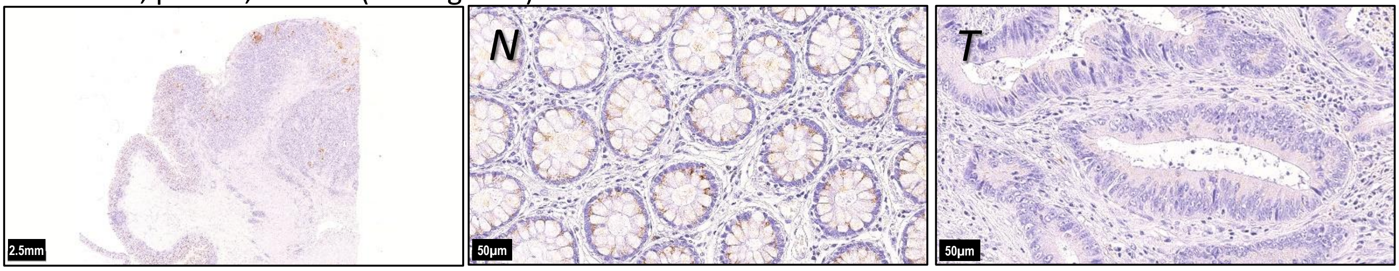

Case 007, dMMR, score 1 (Tn-Negative)

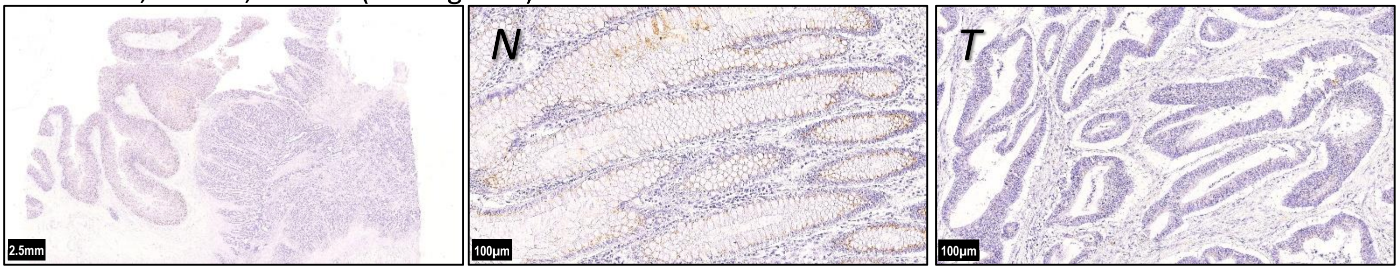

Case 008, dMMR, score 0 (Tn-Negative)

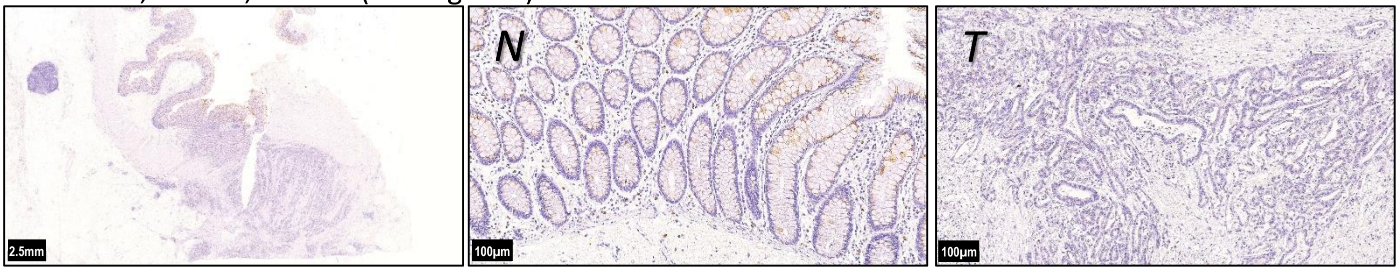

Supplementary Figure S3. Representative cases of tumors showing Tn-Weak (Case 004-005) or Tn-Negative (Case 006-008) expression.

Supplementary Figure S4

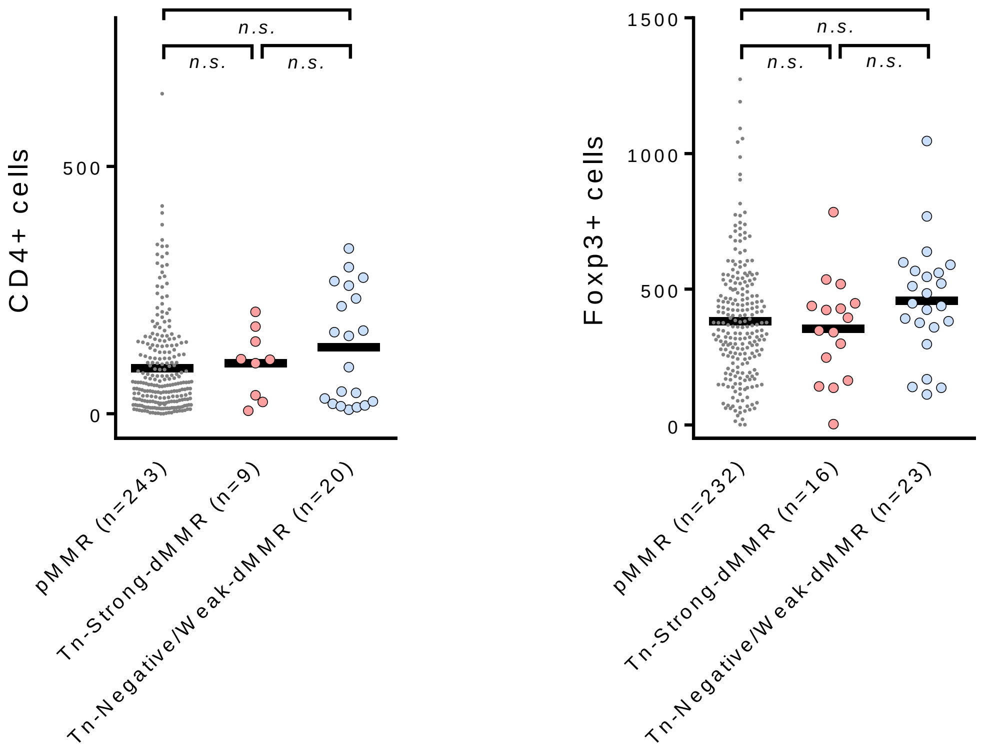

Supplementary Figure S4. CD4+ or Foxp3+ T cell infiltration in pMMR, Tn-Strong-dMMR and Tn-Negative/Weak-dMMR tumors. *n.s.* not significant.

Supplementary Figure S5

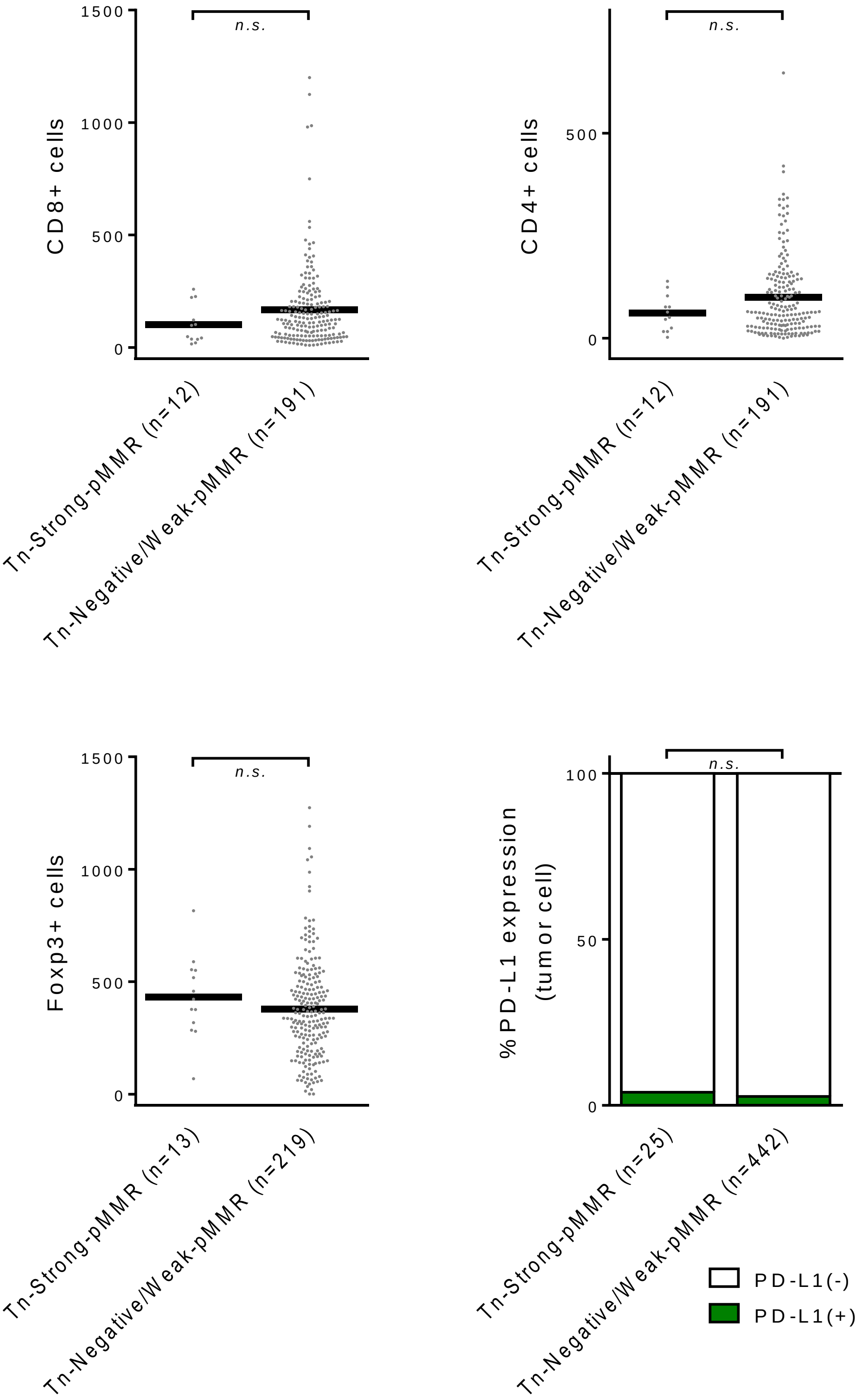

Supplementary Figure S5. CD8+, CD4+ or Foxp3+ T cell infiltration in pMMR tumors, according to Tn antigen expression. *n.s.* not significant.

Supplementary Table 1. Clinicopathological characteristics of colorectal cancer patients according to Tn antigen expression

	Tn-Negative n=189 (37.3%)		Tn-Weak n=277 (54.6%)		*P*
Age					0.369
Mean±SD	68.8±11.0		67.8±11.7		
Gender					0.286
Male	112	(59.3%)	178	(64.3%)	
Female	77	(40.7%)	99	(35.7%)	
Location					0.612
Proximal colon	72	(38.1%)	96	(34.7%)	
Distal colon	48	(25.4%)	78	(28.2%)	
Rectum	69	(36.5%)	103	(37.2%)	
Tumor differentiation					1.000
Well-Moderately	181	(95.8%)	266	(96.0%)	
Poorly	8	(4.2%)	11	(4.0%)	
Histology					**<0.001**
Non-mucinous	189	(100.0%)	262	(94.6%)	
Mucinous	0	(0.0%)	15	(5.4%)	
Tumor invasion					0.435
Tis	9	(4.8%)	21	(7.6%)	
T1	24	(12.7%)	35	(12.6%)	
T2	25	(13.2%)	44	(15.9%)	
T3	78	(41.3%)	99	(35.7%)	
T4	53	(28.0%)	78	(28.2%)	
Lymphatic invasion					0.147
Absent	48	(25.4%)	88	(31.8%)	
Present	141	(74.6%)	189	(68.2%)	
Venous invasion					0.084
Absent	40	(21.2%)	79	(28.5%)	
Present	149	(78.8%)	198	(71.5%)	
Lymph node metastasis					0.382
Absent	122	(64.6%)	166	(59.9%)	
Present	67	(35.4%)	109	(39.6%)	
Not available	0	(0.0%)	2	(0.7%)	
Distant metastasis					0.795
Absent	161	(85.2%)	233	(84.1%)	
Present	28	(14.8%)	44	(15.9%)	
Stage					0.901
0	9	(4.8%)	20	(7.2%)	
I	44	(23.3%)	62	(22.4%)	
II	60	(31.7%)	75	(27.1%)	
III	48	(25.4%)	76	(27.4%)	
IV	28	(14.8%)	44	(15.9%)	
PD-L1 expression on tumor cells					1.000
Negative	179	(94.7%)	262	(94.6%)	
Positive	10	(5.3%)	15	(5.4%)	
MMR status					0.671
pMMR	178	(94.2%)	264	(95.3%)	
dMMR	11	(5.8%)	13	(4.7%)	
CD8+ cells					0.698
Mean±SD	203.5±224.1		193.3±188.8		
CD4+ cells					0.342
Mean±SD	90.2±105.8		102.6±94.0		
Foxp3+ cells					0.908
Mean±SD	384.8±217.9		388.2±232.5		
